# Measuring the Human Ultra-Weak Photon Emission Distribution Using an Electron-Multiplying, Charge-Coupled Device as a Sensor

**DOI:** 10.3390/s18041152

**Published:** 2018-04-10

**Authors:** Fernando Ortega-Ojeda, Matías Calcerrada, Alejandro Ferrero, Joaquín Campos, Carmen Garcia-Ruiz

**Affiliations:** 1 Department of Analytical Chemistry, Physical Chemistry and Chemical Engineering, Multipurpose Building of Chemistry, University of Alcalá, Alcalá de Henares, 28871 Madrid, Spain; fernando.ortega@uah.es (F.O.-O.); matias.calcerrada@uah.es (M.C.); 2 University Institute of Research in Police Sciences (IUICP), Law Faculty, University of Alcalá, Alcalá de Henares, 28801 Madrid, Spain; 3 Institute of Optics “Daza de Valdés”, IO-CSIC, Calle Serrano 121, 28006 Madrid, Spain; alejandro.ferrero@csic.es (A.F.); joaquin.campos@uah.es (J.C.)

**Keywords:** ultra-weak photon emission, spectrometer, measurement, electron multiplying charge coupled device sensor, liquid crystal tunable filter

## Abstract

Ultra-weak photon emission (UPE) is the spontaneous emission from living systems mainly attributed to oxidation reactions, in which reactive oxygen species (ROS) may play a major role. Given the capability of the next-generation electron-multiplying CCD (EMCCD) sensors and the easy use of liquid crystal tunable filters (LCTF), the aim of this work was to explore the potential of a simple UPE spectrometer to measure the UPE from a human hand. Thus, an easy setup was configured based on a dark box for inserting the subject’s hand prior to LCTF as a monochromator and an EMCCD sensor working in the full vertical binning mode (FVB) as a spectra detector. Under controlled conditions, both dark signals and left hand UPE were acquired by registering the UPE intensity at different selected wavelengths (400, 450, 500, 550, 600, 650, and 700 nm) during a period of 10 min each. Then, spurious signals were filtered out by ignoring the pixels whose values were clearly outside of the Gaussian distribution, and the dark signal was subtracted from the subject hand signal. The stepped spectrum with a peak of approximately 880 photons at 500 nm had a shape that agreed somewhat with previous reports, and agrees with previous UPE research that reported UPE from 420 to 570 nm, or 260 to 800 nm, with a range from 1 to 1000 photons s^−1^ cm^−2^. Obtaining the spectral distribution instead of the total intensity of the UPE represents a step forward in this field, as it may provide extra information about a subject’s personal states and relationship with ROS. A new generation of CCD sensors with lower dark signals, and spectrographs with a more uniform spectral transmittance, will open up new possibilities for configuring measuring systems in portable formats.

## 1. Introduction

Biophoton emission or ultra-weak photon emission (UPE) is the spontaneous emission generated by all living systems without an external excitation. Although it was reported that humans could detect direct single photons [1], in practice UPE is not visually detectable in the photopic regime. This is because UPE possesses an intensity of about 10^−16^–10^−18^ W cm^−2^, while the human eye sensitivity ranges from 10^−12^ to 10^−14^ W cm^−2^ [2]. A lot of research began focusing on the measurement of UPE from different in vitro samples once highly sensitive photomultiplier tubes started to be developed [3]. However, huge progress occurred in 1991 when Inaba’s research group developed the first two-dimensional photon-counting device for measuring spontaneous UPE from the human body surface. Authors reported several experiments where human body emitted spontaneous emission under different physiological conditions [4]. After these early findings, many efforts were made on the detection and characterization of UPE from the human body [5]. In fact, UPE has been studied from several viewpoints and proofs of this statement are the recent reviews on the UPE phenomenon considering different aspects like the UPE characterization from the human brain [6] or the effects of traditional Chinese medicine on UPE [7]. Among these topics, the relationship between UPE and cell communication [8,9] is particularly interesting, since it has been demonstrated that cells use photons as information carriers. In 2009 Fels used ciliate *Paramecium caudatum* cultures to test the radiation transmitted between them, confirming that transmission depended on the separation material (glass and quartz were employed) as well as the number of sender/receiving cells (cell density) [10]. Later in 2017, the same author demonstrated for the first time the non-contact physical quorum sensing using the same protozoon [11]. Also, the relationship between UPE and reactive oxygen species (ROS) has been confirmed. Some authors tested the influence of ROS inside differentiated neutrophil-like cells, as well as in the human skin surface and body. It was shown that the UPE increased highly by impregnating the skin surface with peroxides (such as H_2_O_2_), and by enriching the local atmosphere with oxygen [12]. Recently, it has been demonstrated that the recording UPE from HT-29 cells increased with the topical application of H_2_O_2_ as an ROS inducer [13]. Later, HL-60 cells were used for the dynamic monitoring of ROS through UPE detection. UPE was measured for periods of seven days after inducing respiratory burst, and the authors were able to establish a relationship between UPE and oxidative stress metabolism processes [14]. As a consequence, UPE changes have also been proven when inducing oxidative stress processes through physical exercises, such as curl wrist movements of several volunteers [15]. However, the same authors also studied the glutathione reduced and oxidized forms (GSH/GSSG). Surprisingly, despite monitoring a decreasing GSH/GSSG ratio after inducing respiratory burst in HL-60 cells, the change was not statistically correlated to UPE profile, suggesting that computational models are needed for a better understanding of UPE data [16]. 

On the one hand, some external factors such as UPE diurnal and seasonal rhythm [17,18,19], and the influence of external light in terms of delayed light emission [17,20,21], were deeply studied. The latter is of special importance, as light absorbed by the skin and subsequently emitted as delayed luminescence is various orders of magnitude higher than the UPE. Therefore, any exposure to external light should be avoided before attempting to measure spontaneous UPE from the human body. Nakamura et al. stated that the spontaneous UPE decreased when the body temperature decreased [22]. However, while temperature influences spontaneous UPE, they are not directly or linearly correlated—that is, the mechanisms involved in these two processes are differentiable [23]. Moreover, Kobayashi et al. used a CCD sensor to show that thermal images did not match the UPE images [19]. On the other hand, the UPE fluctuations depending on internal factors from the proper subjects were also studied, including the characterization of the UPE pattern of the whole human body [17,24], the influence of age and sex [25,26], the presence of some diseases [24,27,28], or the effect of relaxation techniques [29,30], resulting in less intense UPE in healthy subjects or those practicing relaxation techniques. Even the fact of imagining [31] was proven to have an effect on the UPE intensity.

As a general overview, most of the sensors used to measure the UPE intensity and study different factors affecting it are based on the photomultiplier tubes (PMT) technology, which requires a high-voltage source that makes the experimental setup more complex. Despite obtaining valuable results, the UPE phenomenon is not completely understood. Novel measuring strategies and modern sensing technologies could be useful for further development in this field. Some of these advances have already been reported in the literature. Regarding the measuring strategies, a couple of reports characterized the UPE emission as spectral patterns instead of using intensity as a unique variable [17,28]. Both studies demonstrated that the UPE spectra changed depending on some factors, indicating that recording the spectral pattern and intensity provided extra information about this phenomenon. Regarding the sensing and measuring technology, UPE imaging through charge-coupled device (CCD) sensors was successfully tested in recent years [32]. Ultimately, Kobayashi et al. measured for the first time the UPE spectrum of a human finger by using a CCD sensor instead of a photomultiplier. The authors compared the resulting spectrum and attributed the signal to the melanin emission. However, both spectra were not identical, suggesting the involvement of other mechanisms [33]. 

A new generation of CCD sensors, the electron-multiplying CCD (EMCCD) sensors, offer some interesting features that have not been widely explored in the UPE field. EMCCD present a high sensitivity (based on high quantum efficiency), high performance, great speed, a high dynamic range, and low reading noise. Compared to the CCD, this sensing technology permits amplifying the captured electron signal before it is read out across the camera electronics and the reduction of the effective readout noise [34]. Nonetheless, there is a statistical variation in the overall number of electrons generated from an initial charge packet by the gain register [35] and the gain process goes along with the additional noise from the statistical variation of the gain. As stated previously by Kobayashi et al., this noise from the statistical fluctuation of the gain is a disadvantage when long-time exposure measurements are needed [36], which is a required condition when measuring the human UPE. Additionally, previous reported UPE systems were frequently combined with wavelength controlling means like transmittance diffraction gratings [33] and other sort of band pass filters [17,28]. Several of these devices were large and comprised mechanical components that are relatively slow and may fail during data acquisition. However, a liquid crystal tunable filter (LCTF) has the ability to provide a rapid and vibrationless selection of a reasonably wide tuning range of wavelengths, two-dimensional imaging, a wide field of view, a high switching speed, and large apertures, while keeping the system quite light and very simple. A previous study using an EMCCD sensor focused on imaging the dorsal sides of a subject’s hands [37]. The authors reported a large variability in the emission intensity and pattern, and a high degree of similarity when comparing the left and right hands. Considering the previous literature, this work attempted in a slightly different way the use of the EMCCD sensor and the easy use of a LCTF to measure the UPE spectral distribution of a subject’s hand. This measurement, which is intimately connected with ROS and other biochemical species inside the human organism, could provide relevant information about the physical/personal human state or the presence of different diseases through a non-invasive, high-sensitivity technology.

## 2. Materials and Methods

### 2.1. Instrument Set-Up

**Figure 1:**
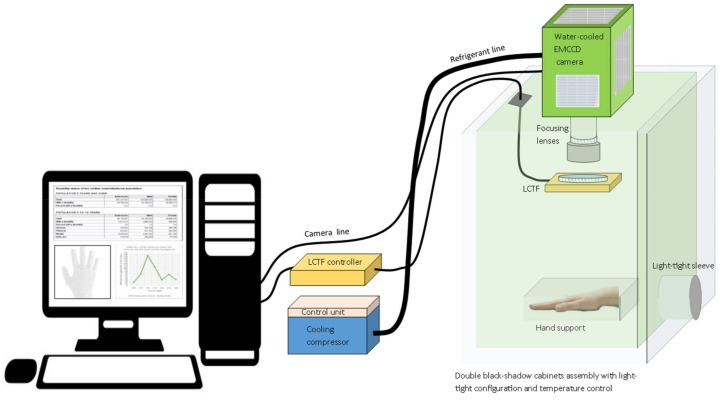
Design of the UPE system for measuring one subject’s hand. The main parts of the UPE system were the EMCCD camera, the focal lens module, the LCTF, the dark chamber, the measuring area for the hand, and the computer. The entire system was placed inside a dark room.

First, an innovative benchtop high-sensitivity UPE spectrometer was set up to perform measurements of the UPE from a subject’s hand. The system included the following components: a next-generation ultra-high-sensitivity cooled camera with an EMCCD sensor (iXon Ultra 888 EMCCD, Andor, Belfast, UK) connected to the top of a dark chamber; a TEC-M55 mm high performance telecentric lens module, comprising an optical objective together with a diaphragm at the objective input (Computar-CBC America, Cary, NC, USA) mounted to the camera from inside the dark chamber; a non-moving-parts LCTF built for the VIS range (VariSpec—PerkinElmer-Cambridge Research & Instrumentation, Waltham, MA, USA) mounted directly in front of the objective lens and in line with it; a control computer for data acquisition and processing; and a dark chamber light-shielded to avoid any light contamination coming from the dark room, where the experiments took place. Figure 1 shows the schematics of the high-sensitivity UPE system for measuring one hand of the subject at a time. The large EMCCD sensor (1024 × 1024) of this spectrometer can capture the wavelength-level variation with high detail. The camera was controlled and the data gathered with the Andor Solis camera software (Andor, UK).

Working as a monochromator, the LCTF was connected to the computer via its serial controller using a RS232/USB interface. The main specifications of this LCTF, which is a variant of a six-stage Lyot filter, are: 7 nm nominal bandwidth (FWHM); 35 mm clear aperture; 400–720 nm spectral range; and 0.01% average out-of-band transmittance. The complete feature list and performance description of this LCTF is given elsewhere [38,39,40]. The individual wavelengths were manually set as needed using a short in-house Matlab script. In other words, the experimental system required the manual set-up of one wavelength at a time prior any UPE reading of the subject’s hand.

The EMCCD camera, with its internal fan always on, was cooled and stabilized at −95 °C using a water bath. This was done in order to reduce the dark current reading and noise, thus increasing the signal to noise ratio and the sensitivity. The pre-amplification gain of the camera was initially kept to a minimum while the exposure time was used to increase the reading’s counts. This is because any noise introduced by the sensor was expected to be amplified by the camera amplifier, thus negatively affecting the reading quality. The camera always used the electron-multiplying type of on-chip amplifier for outputting the readings. For the imaging trials, a tuned built-in setting for highest dynamic range readings was set in the camera: 300–1800 s exposure time; 4.33 μs shift speed; normal vertical clock voltage amplitude; 1 MHz readout rate at 16-bit; pre-amplification gain 1; Binning 1 to 1; and 200–1000 electron multiplying gain. For the spectral distribution trials, the system was set to read in Full Vertical Binning mode (FVB), using only 200 as the electron-multiplying gain necessary for detecting the hand signals. The other EMCCD parameters, except for the exposure time (see below), were the same as for the imaging trials.

The camera and monochromator were located inside a dark box, which had a light-shielded curtain-sleeve working as a front opening for inserting the subject’s hand inside the measuring area of the chamber. It was fundamental to fix the focal distance between the hand and the optical lenses of the UPE system. Hence, the distance and angle of the hand remained the same throughout the measurements by using a hand-support system inside, on the bottom of the chamber for allocating and securing the hand in position. In other words, the hand remained still during the measurements, that is, the system neither accommodates human movement nor measures musculature simultaneously. The field of view of the system covered the four fingers and the knuckles of the subject’s hand. The fingers were hold together because it was shown in very early imaging (non-FVB) trials that the openings between the fingers somehow modified the intensity of the readings.

### 2.2. Subject’s Hand Measurement Procedure

Although this experimental system was set up and tuned with the help of eight volunteers, due to the limitation of the camera availability, the system was finally tested on only one volunteer whose readings were basically complete, replicates included. The subject was a 46-year-old healthy Latin male with a light brown skin color. For the subject’s hand measurement, the following initial conditions were controlled:

(i). The subject’s hand and dark (without hand) signal measurements were carried out inside a dark room,
(ii). Prior to the measurements, the subject wore a long light-tight glove-like sleeve on the selected arm for at least 20 min. Such time would be enough to eliminate any residual delayed luminescence, whilst longer periods would be highly time-consuming for a real application. The glove-like sleeve was made of a double layer of black cotton fabric that was not tight around the arm, thus allowing the hand to breathe without the risk of it getting overheated,
(iii). The external conditions of the dark chamber were controlled, and corresponded to 25 °C external temperature and 45% relative humidity,
(iv). The measurements of the subject’s hand were performed twice for each wavelength, on two consecutive days, and at the same time range for avoiding diurnal rhythms.

Each day’s measurements comprised at most six recordings, which depended on the subject’s availability. Every measurement of the subject’s hand and the dark signal was performed for 10 min at the following wavelengths: 400 nm, 450 nm, 500 nm, 550 nm, 600 nm, 650 nm, and 700 nm. During these measurements, the subject was in complete darkness, and was not forced or induced into any particular emotion but allowed to think and express himself at will. 

The subject was previously informed on the work’s aim and completed a questionnaire (sex, age range, and diseases). All his data were legally treated, preserving anonymity. Furthermore, the subject’s hand measurements were carried out in accordance with the recommendations of the Ethical Committee of the University of Alcalá. Moreover, the measurement protocol was also approved by the Ethical Committee of the University of Alcalá. In addition, the subject was informed and gave written informed consent in accordance with the Declaration of Helsinki.

## 3. Results and Discussion

For the challenging measurement of the UPE from a human hand, two different approaches were tested: imaging and spectral modes. 

When using the imaging mode of the EMCCD camera with tuned built-in highest dynamic range settings, the system rendered non-conclusive 2D count-images of the subject hand. This may be explained by the fact that the EMCCD sensor and camera was designed for very short exposure times, not for the long periods used in this study. In addition, the hand UPE is still too weak to be easily imaged under these conditions, thus its readings did not seem to surpass the overall noise that accumulated and appeared to hide any conclusive image features. That is, the imaging approach and our experimental settings resulted in images with a rather poor signal to noise ratio, which agrees with a previous report on UPE imaging considering these kinds of sensors [36].

**Figure 2:**
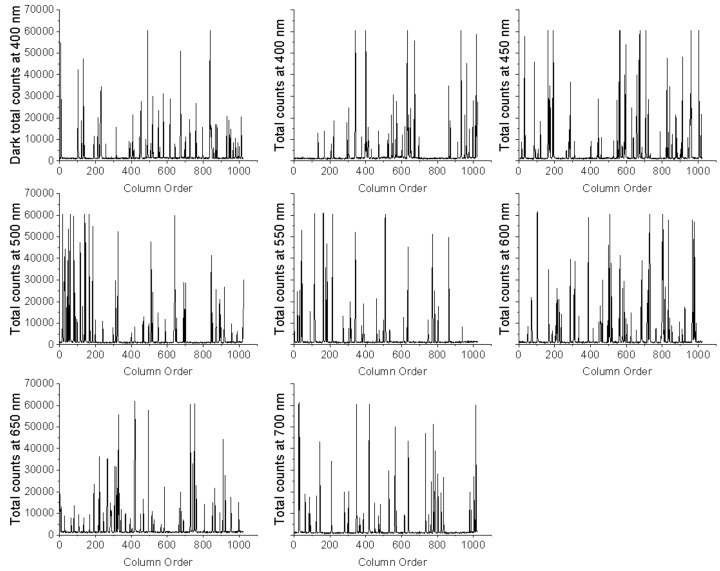
Examples of several FVB images (readings) obtained from the left hand of the subject at various wavelengths.

In order to overcome those non-successful image readings obtained in the EMCCD imaging mode, the EMCCD spectra mode was tested. For this, we used the sensor’s FVB mode because it allows for using the EMCCD-chip as a linear image sensor (a photodiode array). This way, the charge from each column of pixels (each column represents the chip height) is combined (binned) on the chip, yielding a single value per column in a faster way. Figure 2 shows examples of various FVB images (readings) gathered from the left hand of the subject at all wavelengths. Each peak in every image represents the total signal of one column in the sensor. In other words, each peak denotes the accumulation of all the pixels readings at that particular sensor’s column.

**Figure 3:**
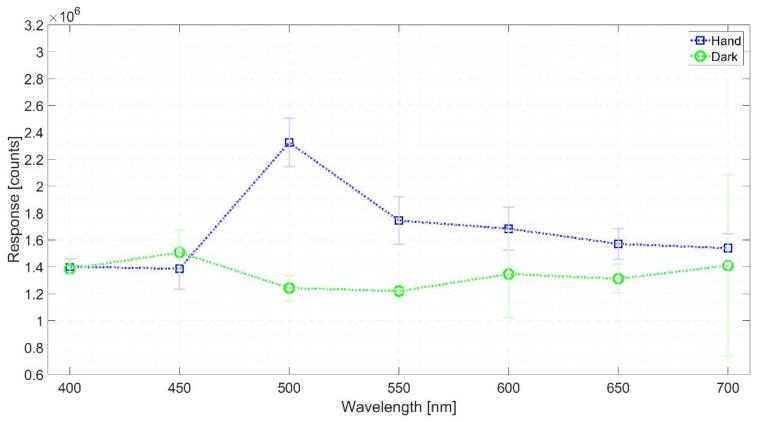
Filtered count vectors depicting the total counts from all the 10-min FVB readings.

The FVB study used 200 as the electron-multiplying gain (EM gain) because previous screening tests (using EM gains from 300 to 1000) indicated that this value was acceptable to render a sufficient hand-dark difference. Moreover, this gain allowed for registering counts while not saturating the sensor at several selected visible wavelengths (450 nm, 500 nm, and 600 nm). The 10-min exposure time was selected because some earlier trials with larger times (15 min to 30 min) either produced saturation in the FVB readings or were uncomfortable for the volunteer, and thus, impractical. As a result, the FVB mode rendered signal vectors whose coordinates correspond to the added readings from each of the EMCCD columns. It was observed that the hand vectors showed a higher count density than the corresponding dark signal vectors. The dark signal results from the combination of the thermally generated electrons and the background photon emission of the LCTF under the supplied voltage. Moreover, both the hand and dark signal vectors had some peaks with comparable intensities, probably due to the spurious noise. The spurious noise or clock-induced charge originates from within the CCD camera when the charge is shifted pixel by pixel towards the output amplifier. In such conditions, there is a very small probability that the charges can knock off additional charges by impact ionization. These unwanted charges generate an additional noise component [41,42]. The large peaks of the dark signal after the long exposure time would have contributed a large amount of noise that probably interfered with the FVB hands UPE readings. Nevertheless, knowing that such a long-exposure EMCCD intrinsic problem must always be considered with this type of sensor, the instrumental parameters ought to be carefully tuned and the signals cleaned. Hence, the spurious signals were cleaned (filtered out) from the FVB readings. This was accomplished in Matlab simply by ignoring the pixels whose values were clearly outside of the Gaussian distribution. Figure 3 shows the total the counts from all FVB vector’s readings once filtered. The red dots correspond to the hand’s reading while the blue ones correspond to the dark signal. With the exception of the 450 nm, on average, the hand count (signal) is comparable to or larger than the dark count. The UPE signals were not seen over the dark signals at the shorter wavelengths, probably because this LCTF has very low transmittance in the blue region (roughly 4%). This is still a positive result in itself, taking into account that the readings were performed with an EMCCD sensor. Except for the value at 700 nm, the variability of the dark counts (in the recording chamber without the subject present) at all the wavelengths was not so large (ca. 128,300 counts). The higher dark reading at 450 nm might be explained by the additional signal coming from the background of the LCTF, which would seem to depend on the wavelength. 

In order to get the system response to the hand UPE, the hand signal count vectors were averaged as well as the dark signals. Afterwards, the dark-averaged vector was subtracted from the hand average vector (hand minus dark FVB signals). The response in counts was converted into emitted photons using Equation (1), which expresses the emitted photons at a specific collection solid angle (given by the distance and aperture of the lens) as a function of the more relevant variables of the detection system. The calculation took into account the spectral quantum efficiency of the EMCCD (the typical values given by the manufacturer), the transmittance of the camera objective (typical values got from a catalogue), and the spectral transmittance of the LCTF, which varies a lot across the visible range [38,39].

(1) $$\mathrm{Emitted}\;\mathrm{photons}\;=\;\frac{(\mathrm{Signal}\;\mathrm{counts}\;-\;\mathrm{dark}\;\mathrm{counts})\;\times \;\mathrm{electron}/\mathrm{counts}\;\mathrm{conversion}\;\mathrm{factor}\;\mathrm{Sensitivity}}{\mathrm{CCD}\;\mathrm{Quantum}\;\mathrm{efficiency}\;\mathrm{at}\;a\;\mathrm{given}\;\mathrm{wavelength}\;\times \;\mathrm{EM}\;\mathrm{Gain}\;\times \;\mathrm{Lens}\;\mathrm{transmittance}\;\times \;\mathrm{LCTF}\;\mathrm{transmittance}}$$

The following values were considered: 16.4 electron/counts conversion factor 1 (electrons per A/D count); 200 EM Gain; 0.50, 0.79, 0.92, 0.98, 0.96, 0.93, and 0.90 External Quantum efficiencies at 400 nm, 450 nm, 500 nm, 550 nm, 600 nm, 650 nm, and 700 nm, respectively; 600 s exposure time; a lens transmittance of 70%; 2.4%, 11%, 26.3%, 31.1%, 35.4%, 39%, and 47.7% of LCTF transmittance at 400 nm, 450 nm, 500 nm, 550 nm, 600 nm, 650 nm, and 700 nm, respectively.

**Figure 4:**
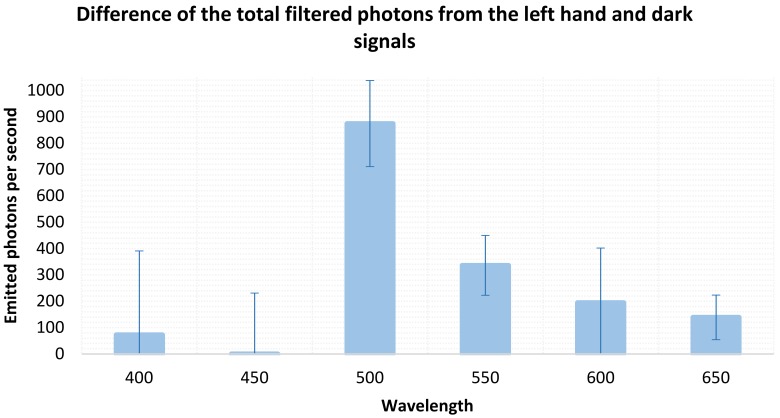
Total filtered FVB readings (photons emitted at the collecting conditions every second) after subtracting the subject’s left hand and dark signal at several wavelengths. Every wavelength was measured for 10 min.

Figure 4 presents the calculated emitted photons at the LCTF spectral bands of 500 nm, 550 nm, 600 nm, 650 nm, and 700 nm where the UPE signal was clearly seen over the dark signal. This work found that the UPE spectral distribution of the subject hand had a peak of about 880 photons at 500 nm. The LCTF’s low transmittance in the blue region might also be a reason for the low emitted photon values found at both 400 nm and 450 nm.

According to Pospíšil et al. [43], several of the reactive oxygen species formed in different cell organelles, and probably involved in the UPE, originate when high-energy intermediates (e.g., dioxetane, tetroxide) decompose. They further generate electronically excited species like triplet-excited carbonyl (3R = O*), emitting in the near UVA and blue–green ranges (350–550 nm); singlet (1P*)- and triplet (3P*)-excited pigments, emitting in the green–red (550–750 nm) and red-near IR (750–1000 nm) ranges; and singlet oxygen (1O2), emitting in the red (634 and 703 nm) and near IR (1270 nm) ranges. The green-emitting ROS might be involved in the UPE signals found at the wavelengths reported in this work.

The spectral distribution found in this work does not agree completely with that for the ventral side of a finger [33], which showed its most prominent region from 500 nm to 650 nm, and peaked at 550 nm and 600 nm. However, the spectral distribution somewhat resembled the central part of the emitted cps from reported right hand fingers [28], showing a maximum UPE emission at 500–550 nm. Moreover, these results are comparable to the UPE research that reported it from 420 to 570 nm [17], or 260 to 800 nm [44], with a range from 1 to 1000 photons s^−1^ cm^−2^ [45].

As a theoretical consideration, the main advantage of EMCCD over PMT is that the former allows for registering the spatial distribution of the incident radiation on the detector, which is impossible in PMT. This advantage, however, disappears when using the FVB mode, and thus the LCTF + EMCCD and LCTF + PMT systems may be considered comparable. Moreover, since both systems have a comparable dark current when working at the same low temperature [46,47], the light response of the EMCCD for the same number of photons would be higher. 

Considering the reported uncertainty of the LCTF [38,39], the difference between the hand and dark signals is statistically significant because it is higher than its uncertainty derived from the readings. Therefore, although these photon numbers are low, this positive result is remarkable, taking into account that it was obtained with an EMCCD sensor and that this particular system was not built for the long exposure required for the UPE measurements.

## 4. Conclusions

In this work, we assembled a simple configuration composed of an EMCCD sensor and an LCTF that allowed for obtaining readings of the UPE from a subject’s hand. It required the use of the sensor in the FVB mode. Despite that, the raw FVB readings from the EMCCD sensor displayed dark signals nearly three times the number of photogenerated electrons Nonetheless, as a global result, with the exception of 450 nm, on average, the hand counts were comparable to or larger than the dark counts. In addition, the UPE spectral distribution of the hand (emitted photons per second) showed a peak of about 880 photons at 500 nm. In that respect, these results agree with some of the research on UPE that reported it from 420 to 570 nm, or 260 to 800 nm, with a range from 1 to 1000 photons s^−1^ cm^−2^. Nonetheless, using an EMCCD sensor in FVB mode and some tuned experimental conditions indeed presents a good possibility for measuring UPE spectral distribution. Given these results, further studies will include a larger number of subjects and the analysis of different factors influencing UPE, such as those linked to ROS enhancement, which could provide information about the personal state of the individual.

Regarding the system reported here and considering constant technology advances, the new generation of CCD sensors (with lower dark signal) and spectrographs (with a more uniform spectral transmittance across the visible range) will open up new possibilities for configuring UPE measuring systems in both portable and benchtop formats. These systems could establish practical measurements of UPE in humans, and relate them to internal body states, which would allow for a deeper understanding of the UPE phenomenon. 

## Abbreviations

cps	counts per second
EMCCD	electron-multiplying, charge-coupled device
FVB	full vertical binning
LCTF	liquid crystal tunable filter
UPE	ultra-weak photon emission
